# Editorial: Bioinformatics, big data and agriculture: a challenge for the future

**DOI:** 10.3389/fpls.2023.1271305

**Published:** 2023-10-16

**Authors:** Sunil Kumar Sahu, Muhammad Waseem, Mehtab Muhammad Aslam

**Affiliations:** ^1^ State Key Laboratory of Agricultural Genomics, Key Laboratory of Genomics, Ministry of Agriculture, BGI Research, Shenzhen, China; ^2^ School of Tropical Agriculture and Forestry (School of Agriculture and Rural Affairs, School of Rural Revitalization), Hainan University, Haikou, Hainan, China; ^3^ Hainan Yazhou Bay Seed Laboratory, Sanya Nanfan Researh, Sanya, China; ^4^ Fang Zhiyuan Academician Team Innovation Center of Hainan Province, Haikou, China; ^5^ School of Life Sciences and State Key Laboratory of Agrobiotechnology, The Chinese University of Hong Kong, Shatin, China; ^6^ College of Agriculture, Food and Natural Resources (CAFNR), Division of Plant Sciences & Technology, University of Missouri, Columbia, MO, United States

**Keywords:** big data, sustainable agriculture, digital agriculture, data analysis, data

The application of bioinformatics and big data analytics to agriculture represents an exciting frontier with an immense potential (Hesami et al., 2022; Khan et al., 2022). These technologies can help drive increased productivity, sustainability, and resilience in agriculture (Sahu and Liu, 2023). However, significant challenges remain in effectively leveraging bioinformatics and big data in the agricultural sector. A key challenge is developing analytical techniques suited for the massive, complex datasets generated by agricultural systems (Aubry, 2019; Hesami et al., 2022). Sophisticated algorithms and methods are required to derive meaningful insights from diverse data sources like genomic sequences, soil nutrient profiles, and climate patterns (Chen et al., 2020; Wang et al., 2023a). Translating these insights into practical solutions will also require an interdisciplinary approach, integrated expertise in biology, computer science, engineering, and social sciences.

Another major obstacle is the lack of data sharing and collaboration in the highly competitive agricultural industry. While proprietary datasets can provide advantages to individual companies, open data platforms and public-private partnerships will be essential for fully realizing the benefits of big data in agriculture (Aubry, 2019; Lawniczak et al., 2022). Improved and efficient data integration and communication across the public and private sectors is critical moving forward. Beyond technical hurdles, deploying big data solutions on a large-scale requires tackling pervasive digital disparities (Aubry, 2019). Subsistence farmers lack of skills in utilizing sophisticated digital technologies. Considerable investments and reform policy are needed to make precision agriculture tools economically viable and accessible to those who need them most. This Research Topic highlights advances in bioinformatics and data science in agriculture in all its diversity, covering both method developments and their applications in agriculture.

The first study by Liu et al. demonstrates the potential of applying bioinformatics and machine learning techniques to analyze agricultural microbiome data for detecting crop diseases. Using a meta-analysis approach, the author(s) integrated microbiome datasets from hundreds of citrus plant samples across different geographic regions and health statuses. They discovered distinct changes in microbial diversity and composition associated with huanglongbing (HLB) disease, identified bacterial biomarkers in the leaves and roots that could accurately predict HLB infection. The random forest and bagging models achieved nearly 100% accuracy in classifying disease states based on select microbiome features. These findings highlight the power of bioinformatics and big data mining to extract valuable insights from complex agricultural datasets. The integration of next-generation sequencing and advanced analytical methods promises to enable early, non-invasive diagnosis of crop diseases like HLB before symptoms manifest.

In the next study, Yang et al. introduce a cloud-based platform called the Variety Test Platform (VTP) to improve the efficiency and quality of crop variety testing. Variety trials are critical for evaluating new crop breeds, but managing the testing process across locations is challenging. The VTP provides tools to standardize trial design, data collection, analysis, and reporting. Key features include configurable trait standards, mobile apps for recording observations, automated design and analysis, and integrated data quality checks. The system was used to manage trials for five major crops across thousands of sites in China. Overall, the integrated platform increased productivity, ensured uniform protocols, and generated shareable, analysis-ready variety of trial data. The VTP demonstrates how modern IT can transform management of the varietal evaluation process to accelerate access to improved crop breeds.

Shen et al. identified and analyzed 26 NHX genes across three *Cucurbita* species - C*. moschata, C. maxima*, and *C. pepo*. Promoter analysis revealed cis-regulatory elements related to salt stress responses. Transcriptome data showed *NHX* genes were differentially expressed under salt stress in *C. moschata* and *C. maxima* leaves. *CmoNHX1* was downregulated with salt treatment. Heterologous expression of CmoNHX1 in Arabidopsis decreased salt tolerance, contrary to expectations. The results provide insights into the evolution and salt stress responses of the NHX gene family in Cucurbita. Overall, the study lays a foundation for breeding salt-tolerant Cucurbita varieties through modulating NHX genes.

Likewise, Wang et al. performed a comprehensive evolutionary analysis of the growth-regulating factor (GRF) gene family in eight important cereal crops. The authors identified 96 GRF genes and found they could be classified into 3 groups based on phylogenetic analysis. Further analysis showed that whole genome/segmental duplications played a major role in the expansion of the GRF family. Expression data revealed GRFs are induced by multiple hormones and are highly expressed in young inflorescences, implying roles in hormone signaling and inflorescence development. Notably, they found that overexpression of one GRF in rice, OjGRF11, increased sensitivity to auxin treatment and inhibited root elongation by altering the expression of auxin signaling genes. Overall, this evolutionary analysis provides important insights into the structure, function and expansion of the GRF gene family in cereal crops.

Looking ahead, bioinformatics and big data will revolutionize agriculture, enhancing productivity, sustainability, and food security globally. Yet fully integrating these emerging technologies into traditional farming requires surmounting systemic challenges. Cross-disciplinary, cooperative initiatives are key to equitably distributing the immense potential rewards. While the promise is vast, patience and nuance will be vital in transitional implementation.

## Author contributions

SS: Conceptualization, Writing – original draft, Writing – review & editing. MW: Writing – review & editing. MA: Writing – review & editing.
